# Co-administration of vancomycin and piperacillin-tazobactam is associated with increased renal dysfunction in adult and pediatric burn patients

**DOI:** 10.1186/s13054-017-1899-3

**Published:** 2017-12-20

**Authors:** Gabriel Hundeshagen, David N. Herndon, Karel D. Capek, Ludwik K. Branski, Charles D. Voigt, Elizabeth A. Killion, Janos Cambiaso-Daniel, Michaela Sljivich, Andrew De Crescenzo, Ronald P. Mlcak, Michael P. Kinsky, Celeste C. Finnerty, William B. Norbury

**Affiliations:** 1 Department of Surgery, University of Texas Medical Branch, 301 University Blvd., Galveston, TX 77555 USA; 2 Shriners Hospitals for Children, 815 Market St., Galveston, TX 77550 USA; 3 Department of Hand, Plastic and Reconstructive Surgery, Burn Trauma Center, BG Trauma Center Ludwigshafen, University of Heidelberg, Ludwig-Guttmann-Str. 13, 67071 Ludwigshafen, Germany; 4 Department of Plastic Surgery, Baylor College of Medicine, 1 Baylor Plaza, Houston, TX 77030 USA; 5 Division of Plastic, Aesthetic and Reconstructive Surgery, Department of Surgery, Medical University of Graz, Graz, Austria; 6 Department of Anesthesiology, University of Texas Medical Branch, 301 University Blvd., Galveston, TX 77555 USA

## Abstract

**Background:**

Burn patients are prone to infections which often necessitate broad antibiotic coverage. Vancomycin is a common antibiotic after burn injury and is administered alone (V), or in combination with imipenem-cilastin (V/IC) or piperacillin-tazobactam (V/PT). Sparse reports indicate that the combination V/PT is associated with increased renal dysfunction. The purpose of this study was to evaluate the short-term impact of the three antibiotic administration types on renal dysfunction.

**Methods:**

All pediatric and adult patients admitted to our centers between 2004 and 2016 with a burn injury were included in this retrospective review if they met the criteria of exposition to either V, V/IC, or V/PT for at least 48 h, had normal baseline creatinine, and no pre-existing renal dysfunction. Creatinine was monitored for 7 days after initial exposure; the absolute and relative increase was calculated, and patient renal outcomes were classified according to the Kidney Disease Improving Global Outcomes (KDIGO) criteria depending on creatinine increases and estimated creatinine clearance. Secondary endpoints (demographic and clinical data, incidences of septicemia, and renal replacement therapy) were analyzed. Antibiotic doses were modeled in logistic and linear multivariable regression models to predict categorical KDIGO events and relative creatinine increase.

**Results:**

Out of 1449 patients who were screened, 718 met the inclusion criteria, 246 were adults, and 472 were children. Between the study cohorts V, V/IC, and V/PT, patient characteristics at admission were comparable. V/PT administration was associated with a statistically higher serum creatinine, and lower creatinine clearance compared to patients receiving V alone or V/IC in adults and children after burn injury. The incidence of KDIGO stages 1, 2, and 3 was higher after V/PT treatment. In children, the incidence of KDIGO stage 3 following administration of V/PT was greater than after V/IC. In adults, the incidence of renal replacement therapy was higher after V/PT compared with V or V/IC. Multivariate modeling demonstrated that V/PT is an independent predictor of renal dysfunction.

**Conclusion:**

Co-administration of vancomycin and piperacillin-tazobactam is associated with increased renal dysfunction in pediatric and adult burn patients when compared to vancomycin alone or vancomycin plus imipenem-cilastin. The mechanism of this increased nephrotoxicity remains elusive and warrants further scientific evaluation.

**Electronic supplementary material:**

The online version of this article (doi:10.1186/s13054-017-1899-3) contains supplementary material, which is available to authorized users.

## Background

Renal failure is a common complication in critically ill patients, particularly in burn patients [1–3]. Acute kidney injury (AKI) that is sustained alongside thermal injury significantly worsens morbidity and mortality in pediatric and adult patients [4, 5]. Over recent decades, the prevalence of multidrug-resistant organisms has been steadily increasing [6, 7], limiting antibiotic treatment options for affected patients and necessitating greater use of aggressive therapeutic antimicrobial combinations [8]. Burn patients are particularly susceptible to infection with multidrug-resistant organisms owing to substantial loss of skin barrier function, wound contamination, nosocomial exposure to pathogens, and impaired post-burn immune function [9–12]. Bacterial infection is a leading cause of death among burn patients [13] and is treated with various combinations of antimicrobials. Vancomycin (V) is a glycopeptide that inhibits cell wall synthesis in gram-positive bacteria; its spectrum of effectivity includes methicillin-resistant *Staphylococcus aureus* [14]. Nephrotoxicity is a side effect of intravenous vancomycin therapy [15]. Imipenem combined with its co-effector cilastin (IC) is a broad-spectrum carbapenem that is commonly used to treat burn-related local and systemic infection caused by *Pseudomonas aeruginosa*, *Klebsiella*, or *Acinetobacter* species [16]. Piperacillin, an extended-spectrum penicillin, combined with the β-lactamase tazobactam (PT) has activity against various gram-positive and gram-negative organisms, including *Pseudomonas* and *Enterobacteria* [17]. Imipenem-cilastin and piperacillin-tazobactam share a similar profile of broad coverage, which enables a degree of interchangeability in burn care [18–20]. Recently, other groups have reported that the combination of vancomycin and piperacillin-tazobactam induces greater renal damage in non-critically ill patients than vancomycin alone [21] or the combination of vancomycin and cefepime [22]. Here, AKI associated with exposure to vancomycin alone (V), vancomycin plus imipenem-cilastin (V/IC), or vancomycin plus piperacillin-tazobactam (V/PT) was quantified based on Kidney Disease Improving Global Outcomes (KDIGO) criteria [23] to determine whether any of these treatment combinations is indicated to be favored regarding nephrotoxicity in adult and pediatric burn patients.

## Methods

### Patients and study design

This study was approved by the Institutional Review Board of the University of Texas Medical Branch (UTMB). As per the Institutional Review Board, this retrospective chart review of de-identified patient data was exempt from additional consenting procedures. A cohort study was designed based on a retrospective chart review between the years 2004 and 2016. All consecutive patients admitted to the UTMB adult burn unit and Shriners Hospitals for Children®—Galveston pediatric burn unit were included in the study if they met the following criteria: admitted for acute burn injury of any size; normal age- and sex-adjusted creatinine at admission; and receipt of either vancomycin alone (V), vancomycin plus imipenem-cilastin (V/IC), or vancomycin plus piperacillin-tazobactam (V/PT) intravenously for at least 48 h of therapeutic treatment. Exclusion criteria were: death upon admission or within the first 48 h following admission; admission for diagnosis other than burn injury; abnormal baseline creatinine; pre-injury renal failure or dialysis; crossing over between the study groups after first exposure to V, V/IC, or V/PT; and incomplete demographic data. Upon admission, burn size and severity were recorded on Lund and Browder charts by the attending surgeon, and demographic data were recorded. If necessary, adult patients were resuscitated as guided by the Parkland formula as previously published [24, 25]. Children were resuscitated according to the standardized Galveston formula as previously published [26, 27]: 5000 ml/m^2^ total body surface area (TBSA) burned + 2000 ml/m^2^ TBSA lactated Ringer’s solution administered in increments over the first 24 h after admission. All patients with full-thickness burns were treated surgically with complete burn wound excision and grafting with auto- or homograft, depending on skin graft availability [26, 27]. In adults, V was administered identically in all study cohorts at 1000–1500 mg every 12 h, and the dose adjusted daily to maintain a trough of 10–15 μg/ml. In children, the starting dose of V was 15 mg/kg every 6 h and the dose was adjusted daily to maintain a trough of 10–15 μg/ml. In adults, 500–1000 mg IC was administered every 6–8 h, with a maximum daily dose of 50 mg/kg or 4 g, whichever was lower. In children, 15–25 mg/kg IC was given every 6 h with daily maximum doses of 2–4 g depending on infection severity. In adults and adolescents > 40 kg, 375–4500 mg PT was administered every 6–8 hours with a maximum 18 g per day; children > 9 months and < 40 kg received 100 mg/kg PT every 6 h.

### Cohorts and endpoints

Patients were allocated to one of the three cohorts based on their exposure to V, V/IC, or V/PT as defined above. Administered doses of antibiotics were recorded and adjusted to patient body weight as the average daily dose (D; mg/kg/day).

For up to 7 days after exposure, serum creatinine (Cr) concentration was monitored daily (a daily average if more than one value per day). The following were determined: baseline concentration (Cr_BL_; mg/dl); maximum concentration (Cr_max_ = maximum value recorded during 7 days post exposition; mg/dl); absolute increase (CrΔ_a_ = Cr_max_ – Cr_BL_ ; mg/dl); and relative increase (CrΔ_%_ = (Cr_max_ – Cr_BL_)/Cr_BL_ × 100).

For the study period, patient status was classified according to the creatinine-based KDIGO stages of acute kidney injury [28]: 1 (≥1.5-fold serum creatinine increase, increase by ≥ 0.3 mg/dL), 2 (≥ 2-fold serum creatinine increase), and 3 (≥ 3-fold serum creatinine increase or ≥ 4 mg/dl).

For 7 days after exposure, daily creatinine clearance was estimated (eCrCl) according to Cr-based formulas. For adult patients, Cockcroft Gault’s formula was used [29]: eCrCl = (140 – age (years)) × bodyweight (kg) × 0.85 (if female)/72 × Cr (mg/dL). For children, the Léger formula was used [30]: eCRCL = (0.641 × weight (kg))/Cr (mg/dL) + (0.00131 × height^2^ (cm^2^))/Cr (mg/dL).

The following were determined: baseline eCrCl (eCrCl_BL_; mg/dl); minimum eCrCl (eCrCl_min_ = minimum value recorded during 7 days post exposition; mg/dl); absolute decrease (eCrClΔ_a_ = eCrCl_min_ – eCrCl_BL_; mg/dl); and relative decrease (eCrClΔ_%_ = (eCrCr_min_ – eCrCl_BL_)/eCrCl_BL_ × 100).

Demographic data, concomitant inhalation injury, and septicemia, as well as occurrence of dialysis throughout hospitalization, were noted.

### Statistical analysis

Continuous, normally distributed data are presented as mean ± standard deviation and were compared using one-way analysis of variance (ANOVA). Nonparametric continuous data were analyzed using the Kruskal-Wallis test. Dichotomous and categorical variables were analyzed using the chi-squared test. A multiple general linear regression model was fit to the continuous response (CrΔ_%_), with average daily dose of V, IC, and PT as independent variables, while adjusting for TBSA percentage burned, age and the presence of inhalation injury. Logistic regression analyses for KDIGO stages 1, 2, and 3 were performed based on antibiotic doses as independent variables. We performed stepwise logistic regression modeling while selecting variables based on minimization of Akaike Information Criterion [31]. Goodness of fit of the logistic regression models was confirmed by the Hosmer-Lemeshow test [32]. Due to the low incidence of AKI events, adult and pediatric patients were analyzed jointly in linear and logistic regression models. As indicated, predictors and responses were transformed for better fitting of model assumptions and the independence of variables confirmed by correlation coefficients. In all comparisons, statistical significance was accepted at *p* < 0.05.

## Results

### Patient characteristics

**Fig. 1:**
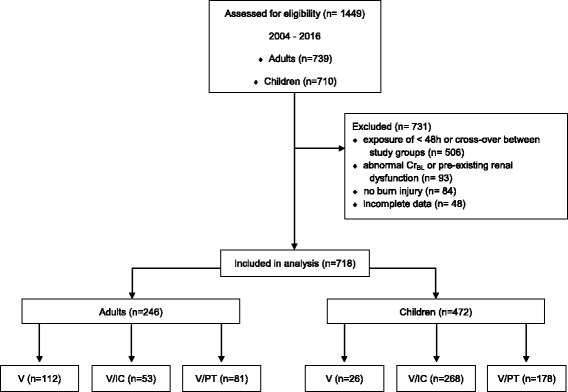
Patient enrollment. *Cr*_*BL*_ baseline creatinine concentration, *V* vancomycin, *V/IC* vancomycin plus imipenem-cilastin, *V/PT* vancomycin plus piperacillin-tazobactam

During the study period, 1449 patients were admitted (739 adults and 710 pediatric patients) to both centers. Figure 1 illustrates the inclusion process into the analysis. Data from 718 (246 adults and 472 children) patients were evaluated. The median sampling time point was the year 2014 for V and V/IC, and 2011 for V/PT.

Characteristics of adult and pediatric patients are shown in Table 1. The study cohorts were comparable with regard to age, sex, TBSA burned, CR_BL_, and incidence of concomitant inhalation injury for both children and adults.

**Table 1:** Patient characteristics at admission

	V	V/IC	V/PT	*p* value
Adults	*n* = 112	*n* = 53	*n* = 81	
Age (years)	46 ± 16	44 ± 16	41 ± 17	0.10
Sex (male:female), *n*	70:39	41:12	78:23	0.09
TBSA (%)	13.5 ± 15	12 ± 12	15 ± 17	0.46
Inhalation injury, *n* (%)	11 (10)	6 (11)	10 (10)	0.90
Cause of burn, *n* (%)				ns
Flame	94 (84)	68 (90)	87 (88)	
Scald	7 (6)	2 (2)	6 (6)	
Electrical	6 (5)	5 (6)	2 (2)	
Other	5 (5)	1 (2)	4 (4)	
CR_BL_ (mg/dl)	0.80 ± 0.18	0.79 ± 0.19	0.81 ± 0.19	0.96
Children	*n* = 26	*n* = 268	*n* = 178	
Age (years)	8 ± 6	7 ± 5	7 ± 6	0.62
Sex (male:female), *n*	17:9	165:103	114:64	0.83
TBSA (%)	32.0 ± 18	39 ± 19	40 ± 18	0.10
Inhalation injury, *n* (%)	3 (12)	48 (18)	19 (11)	0.10
Cause of burn, *n* (%)				ns
Flame	13 (50)	137 (51)	105 (59)	
Scald	7 (26)	102 (38)	57 (32)	
Electrical	2 (8)	24 (9)	11 (6)	
Other	4 (15)	5 (2)	5 (2)	
CR_BL_ (mg/dl)	0.46 ± 0.28	0.50 ± 0.22	0.50 ± 0.21	0.70

Values are shown as mean ± SD unless otherwise indicated

*CR*_*BL*_ baseline creatinine concentration, *IC* imipenem-cilastin, *ns* not significant, *PT* piperacillin-tazobactam, *TBSA* total body surface area burned, *V* vancomycin

### Antibiotic treatment

Study subjects were exposed to antibiotic treatment at comparable times during the course of hospitalization; 93% of V, 98% of V/IC, and 97% of V/PT patients were exposed during the first 24 h after admission (*p* = 0.45). Average daily doses of vancomycin were comparable between cohorts for both children and adults, regardless of administration with IC, PT, or alone. The distribution of days of exposure (2–5 days) was comparable between treatment cohorts for both adults and children, with > 90% of pediatric subjects and > 65% of adults being exposed for 4 or more days. Based on the administration schedules described above, children received lower absolute doses of V, IC, and PT than adults (*p* < 0.0001) but higher average daily doses of V, IC, and PT per body weight (V: 50 ± 17 mg/kg/day; IC: 55 ± 21 mg/kg/day; PT: 257 ± 62 mg/kg/day) than adult patients (V: 21 ± 6 mg/kg/day; IC: 20 ± 6 mg/kg/day; PT: 135 ± 41 mg/kg/day) (*p* < 0.0001).

### Serum creatinine by treatment cohort

#### Adults

**Fig. 2:**
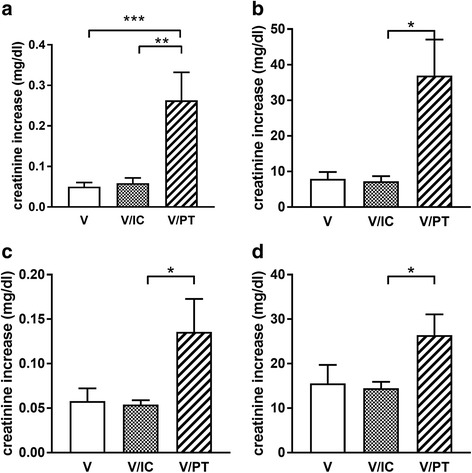
Adult average (**a**) absolute (mg/dl) and (**b**) relative (%) creatinine increase 7 days following exposure to V, V/IC, and V/PT. Child average (**c**) absolute (mg/dl) and (**d**) relative (%) creatinine increase 7 days following exposure to V, V/IC, and V/PT. *p < 0.05, **p < 0.01, ***p < 0.001. *IC* imipenem-cilastin, *PT* piperacillin-tazobactam, *V* vancomycin

During 7 days after exposure to one of the treatments, the average absolute creatinine increase was greater in the V/PT group than in the V or V/IC groups (V/PT: 0.26 ± 0.62 mg/dl; V: 0.05 ± 0.10 mg/dl; V/IC: 0.06 ± 0.09 mg/dl; *p* < 0.001 for V/PT vs. V and *p* < 0.01 for V/PT vs. V/IC; Fig. 2a). No difference was detected between V and V/IC. The average relative increase in creatinine was greater in the V/PT group than in the V or V/IC groups (V/PT: 37 ± 91%; V: 8 ± 20%; V/IC: 7 ± 11%; *p* < 0.001 for V/PT vs. V and *p* < 0.01 for V/PT vs. V/IC; Fig. 2b).

**Fig. 3:**
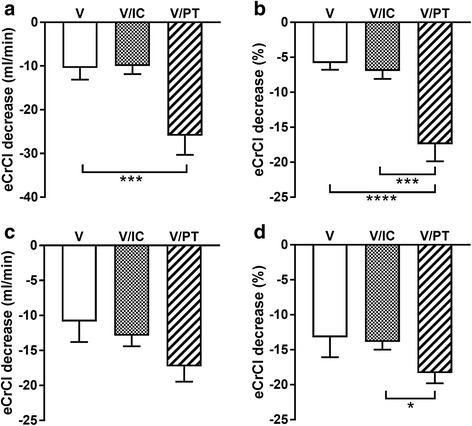
Adult average (**a**) absolute (mg/dl) and (**b**) relative (%) decrease in eCrCl (calculated via Cockcroft-Gault) 7 days following exposure to V, V/IC, and V/PT. Child average (**c**) absolute (mg/dl) and (**d**) relative (%) decrease in eCrCl (calculated via Léger’s formula) 7 days following exposure to V, V/IC, and V/PT. *p < 0.05, ***p < 0.001, ****p < 0.0001. *IC* imipenem-cilastin, *PT* piperacillin-tazobactam, *V* vancomycin

Absolute eCrCl decreased more after exposure to V/PT than to V (V/PT: –26 ± 39 ml/min; V: –10 ± 28 ml/min; *p* < 0.001; Fig. 3a). Relative eCrCl decreased further in the V/PT group than in the V or V/IC groups (V/PT: –17 ± 21%; V/IC: –7 ± 8%; V: 6 ± 10%; *p* < 0.001; Fig. 3b).

**Fig. 4:**
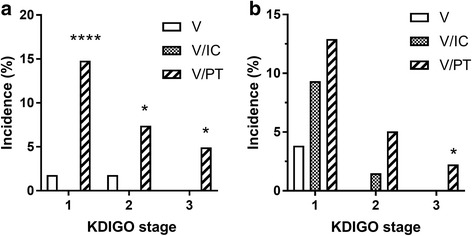
Relative rate of occurrence (%) of AKI events according to KDIGO stage 7 days following exposure to V, V/IC, and V/PT in adults (**a**) and children (**b**). *p < 0.05, ****p < 0.0001. *IC* imipenem-cilastin, *KDIGO* Kidney Disease Improving Global Outcomes, *PT* piperacillin-tazobactam, *V* vancomycin

The rate of occurrence of KDIGO stage 1 was higher in the V/PT group (15%) than in the V (2%) or V/IC group (0%) (*p* < 0.001). KDIGO stage 2 was measured more frequently in the V/PT group (7%) than in the V (2%) or V/IC group (0%) (*p* < 0.05). KDIGO stage 3 of AKI occurred more frequently after exposure to V/PT (5%) compared to V (0%) or V/IC (0%) (*p* < 0.05; Fig. 4a). The adjusted odds ratio of AKI stage 1 for V/PT vs. V/IC was 18.4 (confidence interval 1.06–319.2).

#### Children

Over 7 days of exposure to one of the treatments, the average absolute creatinine increase was greater in the V/PT group than in the V/IC group (V/PT: 0.14 ± 0.49 mg/dl; V/IC: 0.05 ± 0.08 mg/dl; *p* < 0.05; Fig. 2c). No differences were detected between V (V: 0.05 ± 0.07) and V/IC or V and V/PT. The average relative increase in creatinine was greater in the V/PT group than in the V/IC group (V/PT: 26 ± 62%; V/IC: 14 ± 23%; *p* < 0.05; Fig. 2d). No differences were found between V and V/IC or V and V/PT (V: 16 ± 21%).

No differences in absolute decrease of eCrCl were detected between the groups (Fig. 3c). Relative eCrCl decreased further in the V/PT group than in the V/IC group (V/PT: –18 ± 2%; V/IC: –14 ± 18%; *p* < 0.05; Fig. 3d).

The occurrence of KDIGO stage 3 of AKI was higher in the V/PT group (2%) than in the V (0%) or V/IC group (0%) (*p* < 0.05; Fig. 4b). The rate of occurrence of KDIGO stages 1 (V: 4%; V/IC: 9%; V/PT: 13%) or 2 (V: 0%; V/IC: 1%; V/PT: 5%) did not differ between the groups. The odds ratio of AKI stage 3 for V/PT vs. V/IC was 4.01 (confidence interval 1.05–15.4).

### Secondary endpoints

As summarized in Additional file 1: Table S1, length of hospitalization, as well as the incidence of septicemia and mortality, were comparable between the treatment cohorts both in adults and children. However, the incidence of hemodialysis during the course of acute hospitalization was significantly greater in adults treated with V/PT (3%) than in those treated with V or V/IC (both 0%, *p* = 0.03). There was no difference between the pediatric study cohorts regarding the use of renal replacement therapy (RRT). Clinical data of patients who received RRT are summarized in Table 2.

**Table 2:** Clinical characteristics of patients with RRT during hospitalization

Cohort	TBSA (%)	LOS (days)	INH	Septicemia	First drug exposure (days)	RRT begin (days)	RRT duration (days)	Cr_BL_ (mg/dl)	CrΔ_%_ (%)	KDIGO+
Survivors										
V/PT	62	105	N	N	1	6	76	0.8	365	Y
V/PT	80	141	Y	Y	0	40	13	0.7	18	N
V/PT	32	26	N	N	0	10	2	1.01	77	Y
Non-survivors										
V/IC	95	9	Y	Y	1	10	1	0.9	0	N
V/PT	62	234	N	N	0	8	18	0.9	422	Y
V/PT	45	15	N	Y	1	10	4	0.95	21	N
V/PT	40	23	N	Y	0	19	3	0.9	30	N

*Cr*_*BL*_ baseline creatinine concentration, *CrΔ%* relative creatinine increase, *IC* imipenem-cilastin, *INH*presence of inhalation injury at admission, *LOS* length of hospitalization (days), *N* no, *PT* piperacillin-tazobactam,KDIGO+ KDIGO stage 1,2, or 3 during 7 days following exposure, *RRT* renal replacementtherapy, *TBSA* total body surface area burned (%), *V* vancomycin, Y yes

### Statistical modeling

Multivariate models are summarized in Tables 3 and 4. In a joint linear model for the entire patient cohort, while controlling for TBSA, age, and the presence of inhalation injury, the dose of PT had a significant linear effect on CrΔ_%_ (*p* < 0.001). In logistic regression models of all patients combined, after stepwise elimination of covariates, the dose of PT predicted KDIGO stage 1 (*p* = 0.03), 2 (*p* = 0.004), and 3 (*p* = 0.009). There were no significant dose-dependencies for IC or V.

**Table 3:** Multivariable linear regression model of dose dependency of continuous outcome creatinine increase

	CrΔ_%_	
Coefficients	Estimate	*p*
(Intercept)	1.5255	< 0.0001
[D] PT	0.0022	0.0002
TBSA	0.0010	0.7930
INH	0.4103	0.0379
Age	–0.0057	0.1747
[D] V	0.0007	0.8655

All logistic models used stepwise variable selection based on minimization of Akaike Information Criterion and passed the Hosmer Lemeshow test for goodness of fit

*CrΔ*_*%*_ relative creatinine increase (%), *[D] PT* average daily dose of piperacillin-tazobactam (mg/kg/day), *TBSA* total body surface area burned, *INH* presence of inhalation injury at admission, *[D] V* average daily dose of vancomycin co-administered with PT (mg/kg/day)

**Table 4:** Stepwise logistic regression models of dose dependency of categorical outcomes (KDIGO stages 1, 2, and 3)

	KDIGO stage					
	1		2		3	
Coefficients	Estimate	*p*	Estimate	*p*	Estimate	*p*
(Intercept)	–3.9249	< 0.0001	–4.6114	< 0.0001	–4.5883	< 0.0001
[D] PT	0.0036	0.0364	0.0058	0.0039	0.0136	0.0086
[D] V	–	–	–	–	–0.0585	0.1057

All logistic models used stepwise variable selection based on minimization of Akaike Information Criterion and passed the Hosmer Lemeshow test for goodness of fit

*[D] PT* average daily dose of piperacillin-tazobactam (mg/kg/day), *[D] V* average daily dose of vancomycin co-administered with PT (mg/kg/day), *KDIGO* Kidney Disease Improving Global Outcomes

## Discussion

We demonstrate in a large patient cohort that adult and pediatric burn patients sustain independent short-term renal effects of antibiotic treatment with vancomycin plus piperacillin-tazobactam which are not present in patients treated with combinations of imipenem-cilastin and vancomycin or vancomycin alone.

These data are consistent with several smaller reports and case series; studies in diabetic adults with osteomyelitis [33] and heterogeneous non-critically ill patient populations hypothesized that the combination of V/PT induces increased incidences of nephrotoxicity [21, 34]. What is unique about our study design is that we provide a large body of pediatric data and provide a study design that allows for detection of direct effects of drug exposition on selective biomarkers and short-term clinical outcomes. In line with relevant studies, we chose a minimum exposition time of 48 h and an acute follow-up period of 7 days following exposition to detect drug effects independently of other clinical variables which could introduce variance later in the course of burn injury and critical illness. Further, we focused on the established and well-differentiated parameter of serum creatinine per the KDIGO classification to define and detect our endpoints [35, 36].

An increase in serum creatinine is a strong indicator of acute kidney damage in adult and pediatric patients and correlates with acute and chronic renal dysfunction in a graded manner [28, 37–39]. Greater elevations predict the highest risk of morbidity and mortality [40, 41]. Even small CrΔ_%_ ≥ 50% (which corresponds to KDIGO stage 1) double the probability of adverse outcomes and mortality [42]. Our data showed in both adult and pediatric patients that the relative and absolute creatinine increases following exposition to vancomycin/piperacillin were approximately twice those observed with vancomycin/imipenem or vancomycin alone. Accordingly, creatinine clearance was reduced after exposure to vancomycin/piperacillin. Clinically, this corresponded to a significantly increased incidence of short-term renal failure in both adults and children, as well as increased renal risk and injury in adults. The observed effects can likely be attributed to the combination of vancomycin/piperacillin, since the cohorts of V alone and V/IC behaved similarly to each other, as well as in individual comparison to vancomycin/piperacillin. Interestingly, the lower rates of AKI in the vancomycin and vancomycin/imipenem groups did not yield hard clinical advantages in terms of length of hospitalization, septicemia, or mortality. While some of these secondary endpoints tended towards statistical significance, the overall sample size may have been insufficient to reliably detect differences.

In our patient cohort, pediatric patients sustained higher TBSA% burns than adults; therefore, outside of multivariate modeling which reliably corrected for this difference, we analyzed the study groups separately. Our data suggest that the observed effects of antibiotic exposure on the absolute creatinine increase, creatinine clearance decrease, and the incidence of KDIGO events are greater in the pediatric patient population, while differences between the drug groups appear less pronounced. Several factors may contribute to this effect: children were exposed to higher doses of vancomycin, piperacillin, and imipenem when normalized to body weight, which may have caused increased renal damage in itself; the significantly greater severity of injury and associated systemic critical illness, as well as dilution effects of creatinine due to the more aggressive resuscitation measures taken in this group, may have obscured the differences in creatinine increases. Notwithstanding these potentially confounding variables, the relationship between administration of vancomycin/piperacillin and creatinine increase remains significant and robust, indicating that the observed effect is not exclusive to adults.

With regard to endpoints that surpass the 7-day study window, there was an increased incidence of hemodialysis after antibiotic treatment with vancomycin/piperacillin in the adult patient population. Furthermore, all but one patient who progressed to RRT belonged to the vancomycin/piperacillin cohort. However, these results should be interpreted with caution, as they may have been confounded by a multitude of clinical variables during the time after exposure to vancomycin/piperacillin. This is, in part, supported by the fact that only less than half of subjects who progressed to RRT were identified with a KDIGO-positive event during their initial 7-day study period

The mechanistic explanation of our findings remains elusive; nephrotoxicity of vancomycin is thought to be caused by oxidative stress and acute tubular necrosis [43], which in turn can be promoted by product impurities [15], pre-existing renal dysfunction [44], concomitant critical illness [45], and increased doses and duration of administration [21, 46]. Quite recent experimental data by Luque et al [47]. elucidated the matter further by describing a distinct cast-nephropathy caused by nanospheric intratubular vancomycin aggregates in mice and humans (cit). Most clinical studies do not report incidences of vancomycin-associated acute renal dysfunction of more than 5% [45, 46]. Piperacillin-tazobactam in comparison to other β-lactam antibiotics has been associated with impaired renal recovery by some study groups [48], and an additive detrimental effect on renal function in combination with vancomycin has been proposed [21, 49]. It has been suggested that piperacillin may decrease vancomycin clearance, thus leading to increased accumulation and dose-dependent nephrotoxicity of vancomycin [21]. However, this is unlikely to be the case, as comparable doses of vancomycin were administered regardless of the co-administered agent and that administration of vancomycin itself was monitored closely by trough measurements.

Subjects with pre-existing renal conditions were excluded from this study, but it may be concluded that the superimposition of effects of administration of vancomycin/piperacillin on pre-existing acute or chronic kidney injury should be avoided, especially in this at-risk population. Furthermore, the low incidence of KDIGO events in association with vancomycin alone confirms other recent reports [43, 50] which estimate the nephrotoxicity of vancomycin to be lower than commonly described in older literature [51]. Clearly, further research, perhaps in a reliable animal model which allows for analysis of morphologic renal effects, is warranted to elucidate this phenomenon further.

The clinical implications of this study may well extend beyond pediatric and adult burn care. Given the increasing body of evidence suggesting that even small increases in creatinine are indicative of substantially worse outcomes of morbidity and mortality, every effort should be made to reduce nephrotoxicity. Our data suggest that combination therapy of imipenem-cilastin with vancomycin may be advantageous over piperacillin-tazobactam in burn patients, which led to a change in our centers’ clinical practice guidelines in light of the emerging findings of others and this study. However, it needs to be strongly emphasized that replacing piperacillin with imipenem bears great risks in itself, outside potential benefits regarding nephrotoxicity. The increased use of carbapenems has been linked to a substantial increase in multidrug-resistant *P. aeruginosa*, *A. baumanii*, or *S. maltophilia* in various clinical settings and is certainly a concern in burn care [52–54]. Potential ways to mitigate both risks might be to combine vancomycin with other antibiotics with lower resistance-inducing potential or to seek alternatives to high-dose systemic therapy with vancomycin altogether to reduce nephrotoxic potential.

There are several limitations to this study that warrant consideration. The single center, retrospective design precludes inferences which could have been made from a prospectively designed randomized trial. This study focuses on the serum creatinine definition of KDIGO classification as diuresis data was not available in a sufficiently comprehensive manner to be included into the analysis; however, this affects all treatment and age groups equally and should therefore not skew the analysis, despite a tendency to potentially underestimate the incidence of AKI. Furthermore, sensitivity of serum creatinine alone has been reported to be sufficient in detecting AKI and to be more precise than urinary output measures. This study does not have a control group of piperacillin monotherapy to allow for inferences towards whether the observed toxicity of vancomycin/piperacillin is attributable to vancomycin, piperacillin, or both. In light of the large body of evidence that suggests no individual toxicity of piperacillin, future studies will need to verify the exact mechanism underlying the observed phenomenon.

Adjustment for injury severity in this study was based on age, TBSA burned, and the presence of inhalation injury, as these are the most potent predictors in acute burn injury. The lack of established scores such as IGS2 or SOFA as adjustors limits this study’s comparability with other critical care patient collectives outside of burn care.

The group of pediatric patients who received vancomycin alone is disproportionately smaller than the other cohorts. As the children in this trial were more severely burned and had higher incidence of inhalation injury, antibiotic monotherapy was less likely from a clinical standpoint. Furthermore, evaluation of other potentially confounding nephrotoxic agents (such as iodinated contrast agents, aminoglycosides, vasopressors, etc.) administered during the 7 days of monitoring was outside the scope of this study. Because of the high degree of standardization in all aspects of burn injury treatment at our centers, we can assume that potential confounders should affect all treatment cohorts equally and cannot account for observed effects such as the significantly greater creatinine increase averaged over all study patients. The bacteriological indication and efficacy of the antibiotics administered in this study have been described elsewhere [11, 55–58] and were not evaluated to maintain focus on their side effects. Lastly, patients were treated earlier in time with the combination of vancomycin/piperacillin because we discontinued this combination in favor of vancomycin/imipenem when preliminary data became available regarding its potential detrimental effects. While this could in theory skew the analysis towards more positive outcomes due to improvements in care in more recent years, treatment and overall outcomes (such as gross mortality) at our centers have not significantly changed in the median 4 years of time difference between the groups, which makes a confounding effect unlikely. To the contrary, this unintentional “before-and-after” design of this retrospective analysis strengthens its results by equalizing the propensity to receive either V/PT (before) or V/IC (after) for all patients over time and thus preventing potential selection bias.

## Conclusions

The co-administration of vancomycin and piperacillin-tazobactam is associated with disproportionally elevated serum creatinine and lower creatinine clearance when compared to vancomycin alone or its combination with imipenem-cilastin. This increase in serum creatinine is indicative of increased incidence of renal dysfunction based on the KDIGO criteria. The tradeoff between potentially decreased nephrotoxicity of vancomycin/impinenem-cilastin and the risk of induction of multidrug-resistant organisms due to the potential overuse of carbapenems remains a clinical challenge.

## Additional file

Additional file 1: Table S1. Statistical summary of secondary study endpoints. All data are presented as count (percentage) or mean (standard deviation). (DOCX 12 kb)

## Abbreviations

AKI: Acute kidney injury
ANOVA: Analysis of variance
Cr: Creatinine
eCrCl: Estimated creatinine clearance
IC: Imipenem-cilastin
KDIGO: Kidney Disease Improving Global Outcomes
PT: Piperacillin-tazobactam
RRT: Renal replacement therapy
TBSA: Total body surface area
UTMB: University of Texas Medical Branch
V: Vancomycin
