# Supplementary material for: Co-administration of vancomycin and piperacillin-tazobactam is associated with increased renal dysfunction in adult and pediatric burn patients
Source: Crit Care. 2017 Dec 20;21:318. doi: 10.1186/s13054-017-1899-3 (PMC5738705; doi:10.1186/s13054-017-1899-3)
Supplement: Additional file 1: Table S1. — Statistical summary of secondary study endpoints. All data are presented as count (percentage) or mean (standard deviation). (DOCX 12 kb) [file 13054_2017_1899_MOESM1_ESM.docx]

| Secondary study endpoints |  |  |  |  |  |  |  |  |  |
| --- | --- | --- | --- | --- | --- | --- | --- | --- | --- |
|  |  |  |  |  |  |  |  |  |  |
| cohort | adults |  |  |  |  | children |  |  |  |
| treatment group | V | V/IC | V/PT | p |  | V | V/IC | V/PT | p |
|  |  |  |  |  |  |  |  |  |  |
| Length of hospitalization (days; mean (SD)) | 9 (10) | 7 (5) | 8 (8) | > 0.05 |  | 32 (18) | 39 (19) | 40 (18) | > 0.05 |
| Mortality (n, %) | 1 (1) | 0 (0) | 3 (4) | 0.12 |  | 0 (0) | 6 (2) | 1 (1) | 0.29 |
| Septicemia (n, %) | 2 (1.8) | 0 (0) | 4 (5) | 0.16 |  | 3 (12) | 50 (19) | 32 (18) | 0.67 |
| Renal replacement therapy (n, %) | 0 (0) | 0 (0) | 3 (3) | **0.033** |  | 0 (0) | 1 (0.4) | 3 (1.7) | 0.15 |
|  |  |  |  |  |  |  |  |  |  |
